# Structural basis for therapeutic inhibition of influenza A polymerase PB2 subunit

**DOI:** 10.1038/s41598-017-09538-x

**Published:** 2017-08-24

**Authors:** Xiaolei Ma, Lili Xie, Charles Wartchow, Robert Warne, Yongjin Xu, Alexey Rivkin, David Tully, Steven Shia, Kyoko Uehara, Dianna M. Baldwin, Gladys Muiru, Weidong Zhong, Isabel Zaror, Dirksen E. Bussiere, Vincent H. J. Leonard

**Affiliations:** 1 Structural and Biophysical Chemistry, Novartis Institutes for BioMedical Research, Emeryville, CA USA; 2 Protein Sciences, Novartis Institutes for BioMedical Research, Emeryville, CA USA; 3 Virology Lead Discovery, Novartis Institutes for BioMedical Research, Emeryville, CA USA; 4 Global Discovery Chemistry, Novartis Institutes for BioMedical Research, Emeryville, CA USA; 5 Virology, Novartis Institutes for BioMedical Research, Emeryville, CA USA

## Abstract

Influenza virus uses a unique mechanism to initiate viral transcription named cap-snatching. The PB2 subunit of the viral heterotrimeric RNA polymerase binds the cap structure of cellular pre-mRNA to promote its cleavage by the PA subunit. The resulting 11–13 capped oligomer is used by the PB1 polymerase subunit to initiate transcription of viral proteins. VX-787 is an inhibitor of the influenza A virus pre-mRNA cap-binding protein PB2. This clinical stage compound was shown to bind the minimal cap-binding domain of PB2 to inhibit the cap-snatching machinery. However, the binding of this molecule in the context of an extended form of the PB2 subunit has remained elusive. Here we generated a collection of PB2 truncations to identify a PB2 protein representative of its structure in the viral heterotrimeric protein. We present the crystal structure of VX-787 bound to a PB2 construct that recapitulates VX-787's biological antiviral activity *in vitro*. This co-structure reveals more extensive interactions than previously identified and provides insight into the observed resistance profile, affinity, binding kinetics, and conformational rearrangements induced by VX-787.

## Introduction

The influenza A (FluA) and influenza B (FluB) viruses are responsible for seasonal epidemics causing mild to severe respiratory illness with the potential for serious complications in the elderly, infants, and individuals with compromised immune systems. FluA viruses are also characterized by a broad host range, including wild aquatic birds, domestic avian species, and pigs, with potential for transmission from their animal reservoir to humans. These zoonotic transmissions can lead to severe pandemics when novel viruses sustaining human-to-human transmission propagate in a naïve population, as was the case during the last influenza virus pandemic in 2009^1^. The influenza virus genome consists of eight single-stranded RNAs packed into rod-like structures of varying size, known as the ribonucleoprotein complex (RNP)^2^. The conserved 3′ and 5′ ends of each segment are bound to the viral RNA-dependent-RNA polymerase, while the rest of the vRNA is coated with the viral nucleoprotein (NP). These complexes are transcribed and replicated by the heterotrimeric viral polymerase, consisting of PA, PB1, and PB2^3^. Transcription initiation relies on the cap-snatching mechanism, whereby a host pre-mRNA is bound by PB2 through its m^7^G cap^4^ and cleaved by the PA subunit to generate an 11 to 13 bases capped oligomer. This short RNAs are then used to prime mRNA synthesis by the PB1 subunit^5–7^.

Considerable progress has been made in recent years in unravelling the structure and function of the polymerase complexes from various influenza genotypes^8–10^. The central polymerase body is invariantly made of the PB1 subunit, the N-terminal part of PB2 and the C-terminal part of PA. However prominent conformational differences have been noted for the C-terminal part of PB2 and the N-terminal part of PA when comparing different crystal structures. The promoter-bound bat FluA polymerase complex was captured in a “cap-snatching state”, where the PB2 cap-binding domain (309–483, PB2cap) faces PA to promote RNA cleavage^11^. In the promoter-bound human FluB polymerase, PB2cap rotates about 70° and is poised to relay the cleaved host mRNA to PB1, thus representing the “priming of transcription” state^12^. In the absence of the viral promoter, influenza C (FluC) PB2 radically rearranges itself and adopts a “transcription pre-activation” state, where the “closed” cap-binding site occludes capped mRNA binding^13^. Interestingly, this “closed” polymerase conformation is also shared by the 5′ complementary RNA (cRNA)-bound FluB polymerase and the C-terminal two-thirds of PB2 from FluA^14^. Comparison of these influenza polymerase structures reveals a high degree of PB2 plasticity, which appears to be essential to the cap-snatching process^8, 9^.

A few recent breakthrough studies reported the discovery of an azaindole derivative (VX-787) that binds the PB2 cap-binding site and inhibit FluA replication by blocking viral transcription^15, 16^. The co-structure of VX-787 with the PB2cap has been reported by Clark *et al*.^16^, showing several key interactions within the known cap-binding site. Nevertheless, the mode of action of VX-787 is not fully explained by this co-structure. Clark *et al*. noted that the carboxylic group of the ligand may form additional direct interactions with PB2 owing to its substantial contribution to the binding affinity^16^. Moreover, resistance studies identified not only residues in the cap-binding pocket (Q306, S324, F404), but also a mutation outside PB2cap (N510T) associated with >130 fold resistance to VX-787^15^. This may suggest an incompletely defined VX-787 binding pocket or an allosteric effect.

Here, we describe the discovery and characterization of PB2long (FluA H3N2 PB2 241–741), an extended PB2 protein construct that more completely represents native PB2 cap-binding site. Structural characterization of the PB2long/VX-787 complex for the first time reveals new structural determinants of PB2 antagonist binding that are outside the known cap-binding domain. The functional importance of these additional interactions at the “mid-linker” region was further corroborated by biochemical assay, biophysical characterization and mutagenesis studies with resistant mutant N510T. Finally, this closed state of the PB2 binding site resembles those in m^7^GTP bound FluA PB2-C, the apo-FluC polymerase and cRNA-bound FluB polymerase, and thus provides insights into the conformational selection of PB2 by an antagonist.

## Results and Discussion

### Identification of PB2long

**Figure 1:**
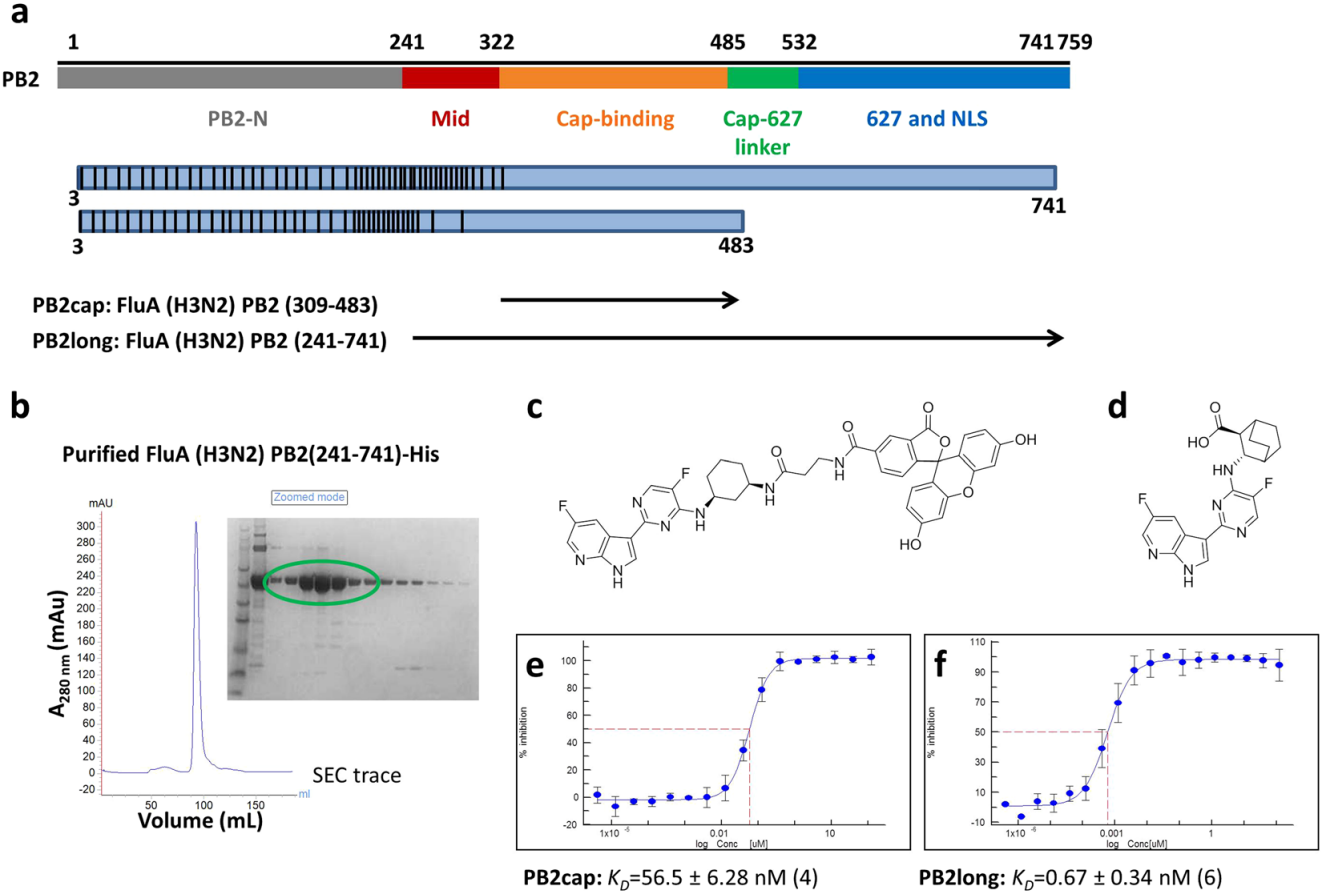
Protein engineering and FP binding properties of H3N2 PB2long. (**a**) Schematic representation of the high-throughput cloning of PB2. The black vertical line represents the starting position of each truncation, ranging from amino acid 3 to 318. The C-terminal amino acids are indicated at the end of each blue rectangle box. The black arrows represent the final constructs that were used in this study. (**b**) The elution profile of H3N2 PB2long-His purified by size-exclusion chromatography (SEC) and Coomassie blue-stained SDS-PAGE of the protein. Fluorescein (FAM)-labelled VX-787 analog (**c**) was incubated with increasing amounts of PB2 protein. The affinity of VX-787 (**d**) towards FluA PB2cap and PB2long measured by fluorescence polarization. Error bars, standard deviation from four or six independent measurements for PB2cap (**e**) and PB2long (**f**), respectively.

When examining ligand binding to PB2cap by fluorescence polarization (FP) assay (Fig. 1a,c), we found that the dissociation constant of VX-787(*K*_*D*_ = *56.5 nM*) was twenty-fold weaker than its potent antiviral activity in the cytopathic effect assay using the A/Udorn/72/H3N2 strain (EC_50_ = 2.5 nM, n = 3, Fig. 1d,e). This large disconnect between the *in vitro* binding activity and the cellular activity led us to hypothesize that PB2cap encodes an incomplete cap-binding pocket for the inhibitor. To better understand the determinants of PB2 inhibition, we set out to identify a construct more representative of the protein in the heterotrimeric complex by extending the domain boundaries of PB2cap, even though multiple random library screens of PB2 construct were attempted^4, 17–19^. Taking a semi-rational design approach, two C-terminal positions were chosen for the majority of the FluA PB2 constructs: position 741, excluding the last 18 residues of the nuclear localization sequence^19^, and position 483, marking the ending of PB2cap (Fig. 1a and Supplementary Fig. S1a). The N-terminal positions range from the 3rd to 318th in the amino acid sequence, with gaps of 2–20 residues in between. Among the 93 constructs generated, a number of truncations showed good expression in the small scale expression screening (Supplementary Fig. S1b). To identify longer PB2 constructs, we cherry picked several clones ending at 741 for solubility and purification evaluation (Supplementary Table S1). The PB2 construct encompassing residues 241–741 (PB2long) demonstrated the highest solubility and yield (15 mg/L) and was therefore selected for further characterization (Fig. 1b).

The FP binding assay with PB2long revealed that VX-787 bound to PB2long with a *K*_*D*_ of 0.67 nM, which is more than 80-fold higher affinity than PB2cap binding and in agreement with the antiviral activity of VX-787 *in vitro* (Fig. 1f). The pronounced differences between the binding of VX-787 to PB2long and PB2cap suggest that structural elements outside the known cap-binding domain also contribute to VX-787 binding directly or allosterically. These results prompted us to carry out further structural and biophysical studies on PB2long to gain additional insights into its ligand binding properties.

### Crystal structures of PB2long

**Figure 2:**
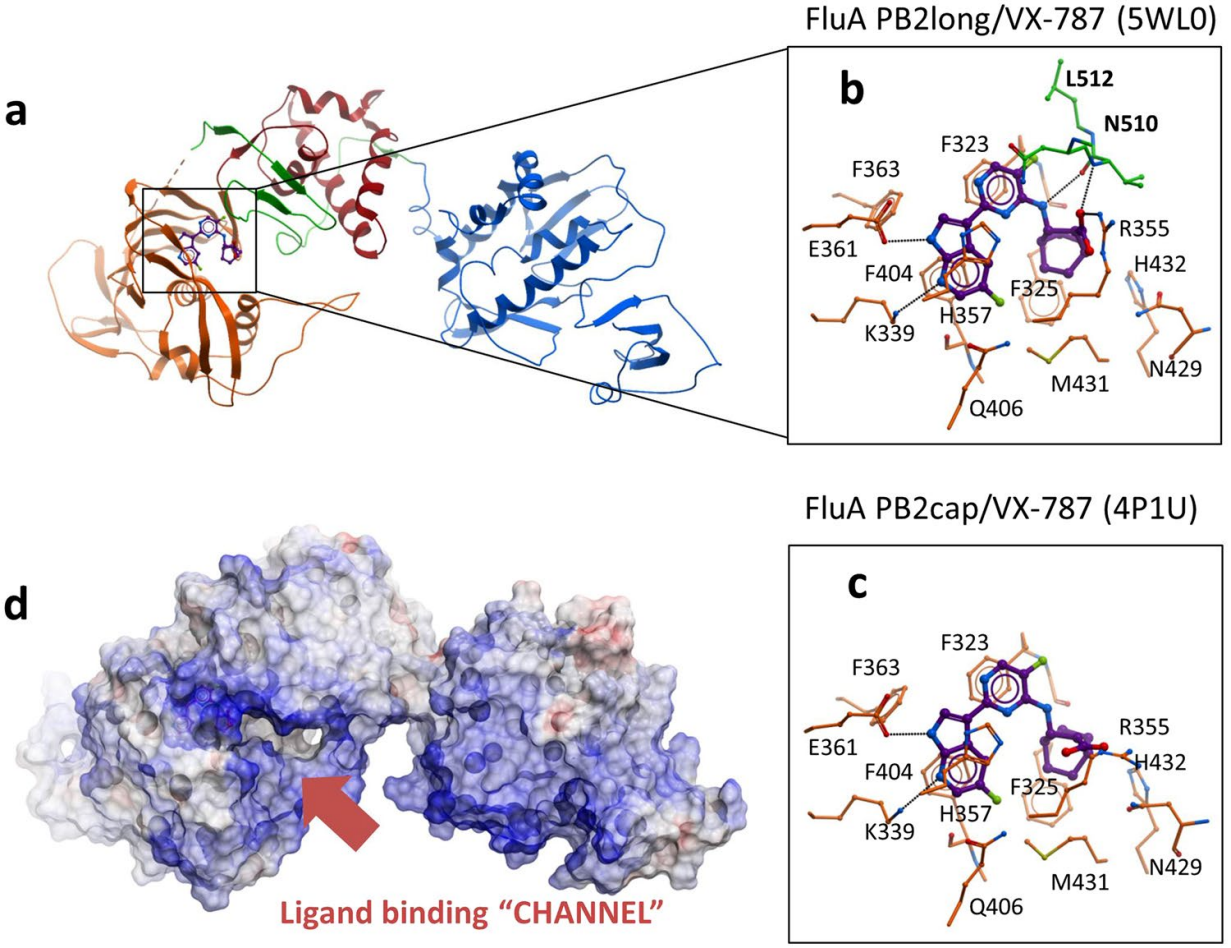
Structural characterization of FluA PB2long. (**a**) Crystal structure of PB2long/VX-787 and detailed interactions of VX-787 with PB2long (**b**) or PB2cap (**c**, pdb accession 4P1U). (**d**) Electrostatic surface of PB2long with bound VX-787.

**Figure 3:**
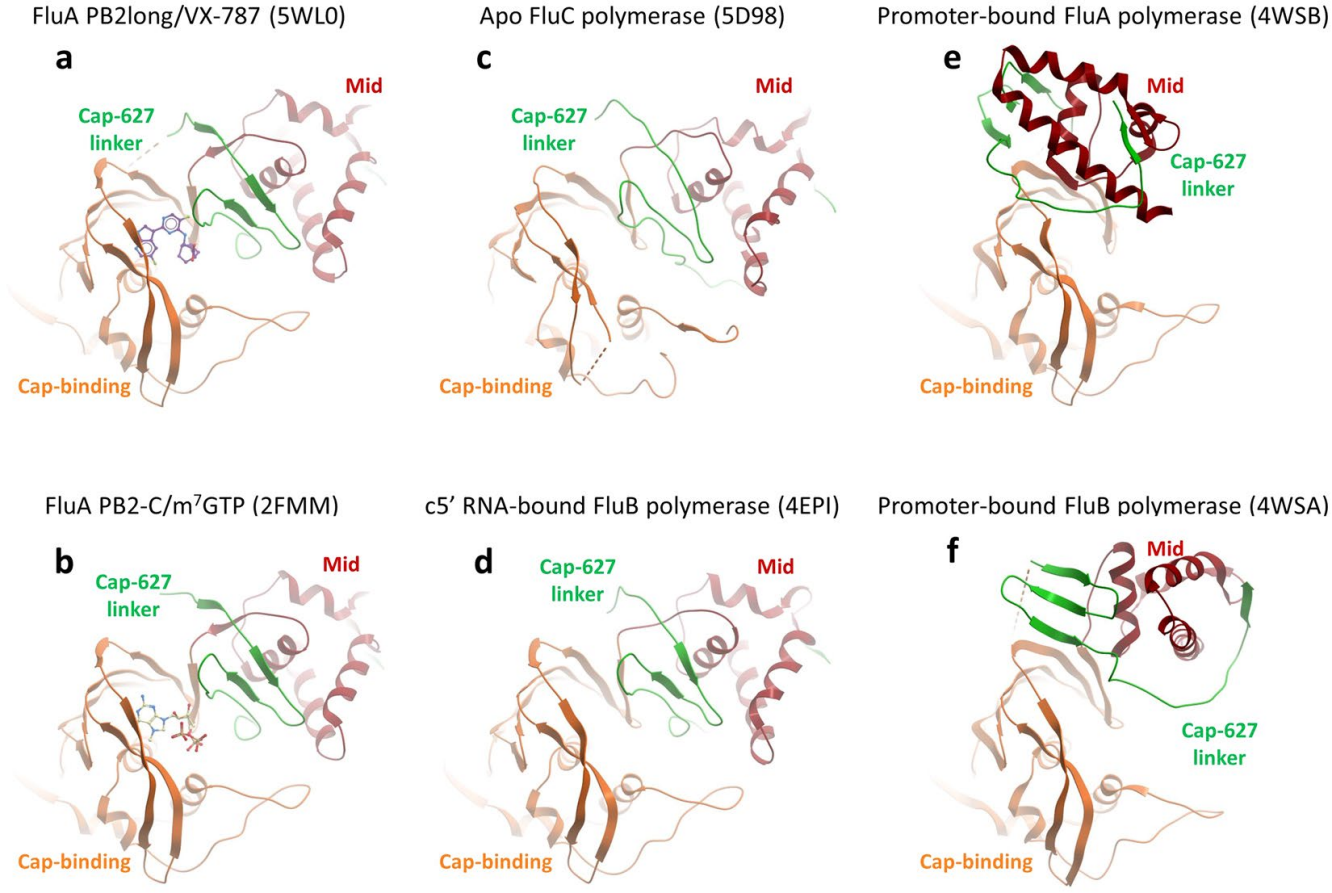
VX-787 targets the transcription pre-activation state of FluA PB2. Comparison of PB2 Mid/Cap-binding/Cap-627 linker in (**a**) VX-787-bound FluA PB2long (this work); (**b**) m7GTP-bound FluA PB2-C (pdb accession 2FMM); (**c**) Apo FluC polymerase (pdb accession 5D98); (**d**) 5′c RNA-bound FluB polymerase (pdb accession 4EPI); (**e**) promoter-bound FluA polymerase (pdb accession 4WSB); (**f**) promoter-bound FluB polymerase (pdb accession 4WSA).

Next, we determined the co-crystal structures of PB2long complexed with VX-787 at 2.4 Å (Table 1, Fig. 2a and Supplementary Fig. S2). Overall, the cap-binding domain in PB2long is similar to the previously determined structure of PB2cap bound to m^7^GTP (r.m.s. deviation of 0.86 Å for main-chain atoms)^4^ and to the cap-binding domain embedded in the influenza A polymerase complex (r.m.s. deviation of 0.85 Å for main-chain)^11^. However, the arrangement of domains flanking the cap-binding region is drastically altered in our structures compared to the influenza A polymerase trimer (Fig. 2a and Fig. 3a,e). The N-terminal and C-terminal extensions of the minimal cap-binding domain come together and fold into a novel functional domain termed “mid-linker” domain^14^. Although the mid-linker domain consists of discontinuous segments, it moves as a rigid body and is positioned adjacent to the PB2 cap-binding site in the PB2long structure. The hinge for this movement is anchored at the junctions between 321–324 and 484–498, and was previously described by comparing the influenza A and B polymerase structures^12^. Furthermore, the RNA-binding and nuclear localization sequence (NLS) domain assumes an elongated, linear conformation rather than the folded-back conformation as in the promoter-bound FluA polymerase complex^11^. Thierry *et al*. recently described a similar PB2 construct 247–736 of A/Vietnam/1203/2004(H5N1) to determine the structure of PB2-C in the unbound and m^7^GTP-bound state, and found that the PB2-C radically repacks itself compared to that observed in the FluA and FluB promoter-bound structures^14^. Superposition of this model to the VX-787 bound PB2long shows that the overall features and architecture of this region of PB2 in isolation are largely similar (r.m.s. deviation of 1.26 Å for main-chain atoms, Fig. 3b), thus lending support to the conserved nature for such PB2 domain arrangement.

**Table 1:** Data collection and refinement statistics.

Data collection	VX-787 complex
PDB ID	5WL0
Resolution (Å)	43.00–2.40 (2.49–2.40)*
Space group	C2
**Cell dimension**	
a, b, c (Å)	142.41, 56.75, 82.01
α, β, γ (°)	90.00, 111.23, 90.00
Total reflections	90471 (9043)
Unique reflections	24138 (2401)
Multiplicity	3.7 (3.8)
Completeness (%)	99 (100)
I/σI	13.07 (1.63)
R_merge_	0.09619 (1.001)
CC1/2	0.997 (0.678)
CC*	0.999 (0.899)
**Refinement**	
R_work_	0.2012 (0.2990)
R_free_	0.2507 (0.3763)
Number of non-H atoms	3874
macromolecules	3791
ligands	29
Protein residues	482
RMS (bonds)	0.008
RMS (angles)	1.08
Ramachandran favored (%)	96
Ramachandran allowed (%)	4
Ramachandran outliers (%)	0
Clashscore	10.01
Average B-factor	63.75
macromolecules	63.96
ligands	49.41
solvent	56.59

*Values in parentheses are for highest-resolution shell. A single crystal was used for this structure. **R_work_ and R_free_ were calculated from the working and test reflection sets, respectively. The test set contained 5% of the total reflections available.

### Structural comparison of VX787-bound PB2long and PB2cap

In comparison to the previously reported co-structure of VX-787 with PB2cap^4^, many of the key interactions are well maintained in PB2long: the azaindole core is positioned between two aromatic residues, H357 and F404, and firmly anchored by a pair of H-bonds to K376 and E361, while a second pi-stacking interaction is observed between pyrimidine and F323 (Fig. 2b,c). However, one consequence of the extended mid-linker region in PB2long is that residues 520–535 impinge on the PB2 cap-binding site and directly interact with the ligand, thereby revealing new determinants of VX-787 binding. The carboxylic acid of VX-787 was previously shown to be a key contributor to PB2 binding^20^. In the PB2cap/VX-787 complex this moiety is solvent exposed and forms water-mediated interactions with H357, Q406, and R355 (Fig. 2c). Direct interactions with neighboring residues, such as K339 and R355, were suspected but not directly observed. In the PB2long/VX-787 complex, the carboxylic acid of VX-787 rotates approximately 90° to form electrostatic interactions with the side chain of R355 and a hydrogen bond to the main chain amide of N510 (Fig. 2b). Moreover, the mid-linker region forms additional interactions with VX-787 by contributing a hydrogen bond to the amine group and several van der Waals contacts (from N510, V511 and L512). These additional interactions not only explain why VX-787 has higher binding affinity to PB2long than to PB2cap, but also rationalize the substantial improvement in binding affinity after installing the carboxylic group.

### SPR binding characterization of PB2 variants

**Figure 4:**
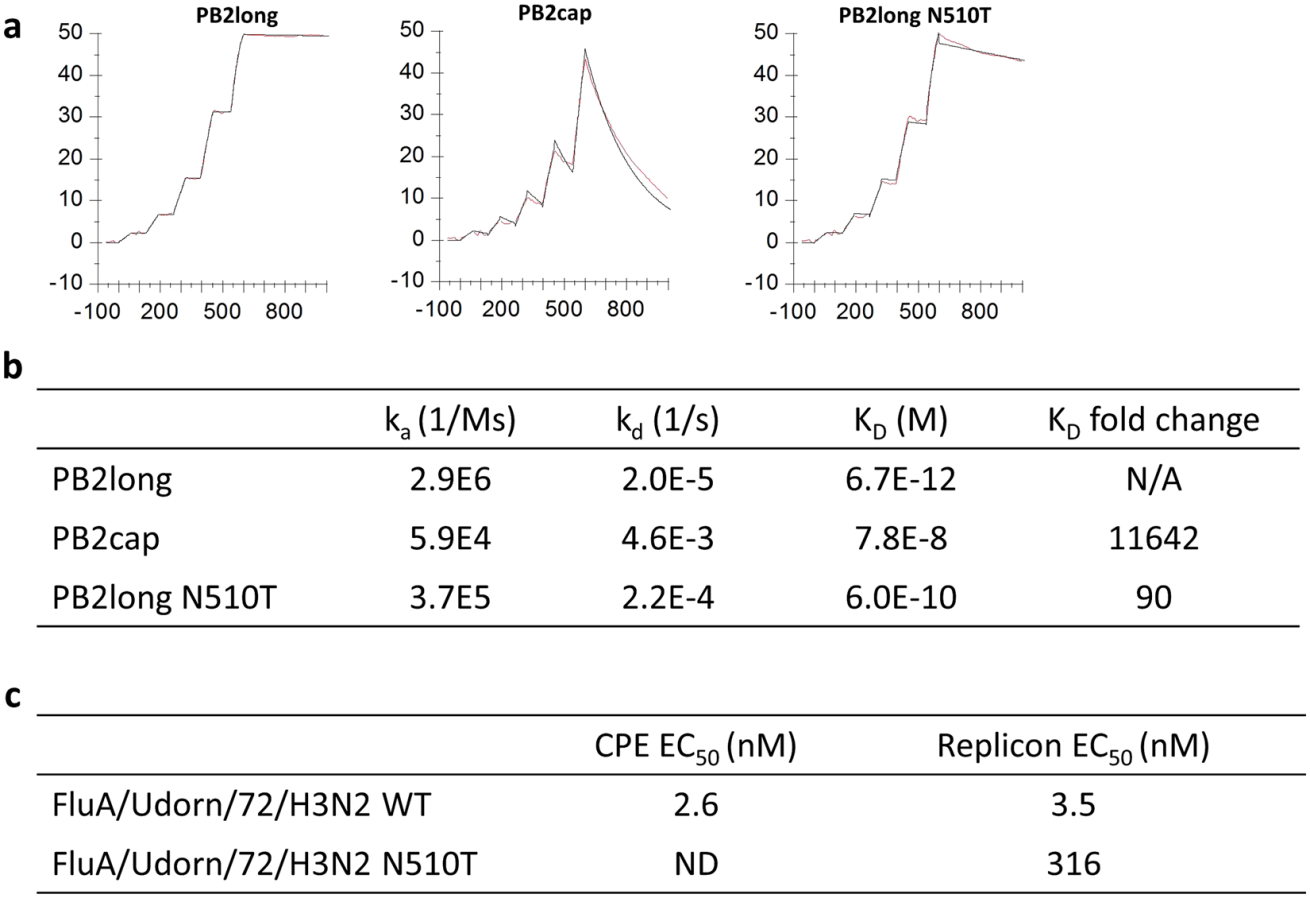
Comparison of FluA PB2long, PB2cap and PB2long N510T mutant by SPR binding assay and cell activity assays. (**a**) Single-cycle SPR sensorgrams for interactions of VX-787 with PB2long (left), PB2cap (middle) and PB2long N510T mutant (right). (**b**) Association (K_a_) and dissociation rates (K_d_), dissociation constant (K_D_), and fold change in K_D_ of VX-787 with three PB2 forms in the SPR assay. (**c**) Cellular activity of VX-787 in the CPE assay and EC_50_ shift in the replicon assay between wild type and the N510T PB2 proteins. Range of concentration tested: 100 μM to 3.16 pM, 16-point dilutions. ND: not determined.

The binding affinity and kinetics of VX-787 with PB2long, PB2cap and PB2long N510T mutant were investigated by surface plasmon resonance (SPR). The affinity of VX-787 for PB2long was more than three orders of magnitude greater than for PB2cap, mainly due to a very slow off-rate (Fig. 4a,b). This affinity was also significantly higher than the K_D_ from the FP results, which may be due to the lower limit of the FP assay (~1 nM). Reduced off-rates, which lead to a longer “resident time” of a compound bound to its target, have been described as a key feature of many marketed drugs^21^. In the case of PB2cap, the ligand is positioned at the surface of the truncated binding pocket, exposed to the periphery of the bulk solvent and has fewer beneficial enthalpic interactions with the protein. This leads to fast-on, fast-off binding kinetics. In PB2long, the ligand is more deeply positioned in the binding pocket and forms more beneficial enthalpic interactions with the protein (Fig. 2d); thus, the ligand is largely shielded from bulk-solvent by the protein. This closed binding mode likely contributes to the slow inhibitor off-rate and a longer half-life of this complex. Moreover, PB2long N510T point mutant resulted in a 90-fold decrease in SPR binding affinity for VX-787 (Fig. 4b). This result is consistent with our structural observation that this residue is adjacent to VX-787 in the PB2long structure (Fig. 2b), where the sidechain nitrogen of the asparagine directly interacts with the pyrimidine of the compound via a cation-π interaction. Replacing this favorable interaction with a bulky Cβ branched threonine would likely introduce unfavorable steric clashes with the compound, consequently reducing binding affinity. Furthermore, the functional significance of this residue was also confirmed in our influenza virus replicon assay. The EC_50_ generated for VX-787 in the WT replicon assay (A/Udorn/1972 EC_50_ = 3.5 nM) is consistent with CPE data generated with the same viral strain (EC_50_ = 2.6 nM, Fig. 4c). We therefore generated the substitution N510T in the PB2 protein of the FluA Udorn replicon and compared its activity to the wild type protein. The activity of PB2 N510T in the replicon assay was similar to the activity of the WT protein, suggesting no loss of fitness at the replication level for this mutation. In presence of VX-787, the substitution resulted in a 90 fold losses of cellular EC_50_ (Fig. 4c) confirming the importance of PB2 N510 in the context of the full replication complex.

### Conformational selection in the recognition of VX-787

The PB2 structures, reported here and previously, reveal a considerable structural plasticity even in the crystalline state^9, 10^. The PB2 subunit can adopt dramatically different conformations depending on the type of ligand bound. Compared to the “cap-snatching state” in bat FluA or the “priming of transcription state” in human FluB (Fig. 3e,f), VX-787 binding appears to require rotation of PB2cap to clamp onto the middle domain (Fig. 3a). As a consequence of this conformational change, the PB2 cap-binding site, which is housed between the cap-binding domain and the neighboring mid-linker domain, is completely reshaped and consequently no longer accessible (Fig. 2d). Most notably, this closed state was captured not only in the m^7^GTP bound FluA H5N1 PB2-C but also in the apo-FluC polymerase and cRNA-bound FluB polymerase (Fig. 3b–d)^13, 14^.

Two distinct mechanisms have been proposed to address ligand-mediated conformational changes: induced fit and conformational selection^22–24^. In the latter paradigm, a protein samples multiple conformational states, including the ligand-bound state. The PB2 domain arrangement in PB2long/VX-787 mirrors those seen in the m^7^GTP bound FluA H5N1 PB2-C, the apo FluC and c5′-RNA bound FluB polymerases, indicating that this structure is common for the ligands. This is in line with the conformational selection model of molecular recognition, which assumes the pre-existence of multiple conformations of the macromolecule from which the ligand selects the optimal one. The presence of VX-787 most likely favors the closed PB2 state over the other accessible states due to conformational selection, thus trapping the enzyme in the “transcription pre-activation” state.

## Conclusion

In summary, we have identified a stable, soluble PB2 construct PB2long that has improved functionality compared to the minimal cap-binding domain PB2cap. Structural characterization of PB2long revealed an independently folded mid-linker that makes additional contacts with VX-787, completing the PB2 cap-binding site by partially occluding the ligand from solvent. In line with this structural observation, solution biophysical studies of VX-787 with PB2long exhibit exquisite high potency and an extremely slow off rate. The resistance mutation PB2^N510T^ illustrates the relevance of this residue outside the cap-binding region in engaging VX-787 and supports the hypothesis that FluA polymerase can adopt a conserved, closed PB2 conformation. A similar closed cap-binding site has been observed for m^7^GTP bound FluA PB2-C, and full-length PB2 protein in the apo FluC polymerase and cRNA-bound FluB polymerase^14^. We therefore propose that VX-787 favors the closed PB2 cap-binding site, trapping the enzyme in the “transcription pre-activation” state.

## Methods

### Identification of PB2long by high throughput cloning

The DNA encoding sequence of PB2 subunit of influenza A/Udorn/307/1972(H3N2) was *E. coli* codon optimized and synthesized by Genewiz. The gene was cloned into pTrcHis2b vector (Thermo Fisher Scientific), resulting in a full-length PB2 protein with a C-terminal His6-tag. High-throughput cloning was carried out using the full-length PB2 plasmid as the template, the corresponding primers (Supplementary Table S2), and Q5 site direct mutagenesis kit (NEB) to generate 96 distinct PB2 constructs with various lengths. 93 constructs were obtained and their sequences were confirmed (Supplementary Fig. S1a **)**. The plasmids were transformed into *E. coli* BL21(DE3) cells. Each clone was grown in 4 mL terrific broth (TB) in the 24-well plate (4 plates total). The cells were induced with 1 mM IPTG when OD reached 0.6–1, and continued to grow at 18 °C overnight. Cells were harvested the next day and the total lysates were analyzed by SDS-PAGE (Supplementary Fig. S1b). Several long constructs (A6, A9, A12, B5, C3, C9, C12, D2, D4, D5, D6) were cherry picked for 1 L expression. Following the same procedure as below, the cells were lysed by sonication and subject to HisTrap HP column purification, which led to the identification of PB2(241–741) (PB2long, D2, Supplementary Table S1).

### Protein expression and purification

*E. coli* BL21(DE3) cells harboring pTrcHis2b-PB2(241–741)-His plasmid were grown in terrific broth. The cells were induced with 1 mM IPTG when OD reached 0.8, and continued to grow at 18 °C overnight. The cells were harvested on the next day and stored at −80 °C until usage. Cell pellets were resuspended in the lysis buffer (20 mM Tris, pH 8.0, 500 mM NaCl, 1 mM TCEP, 20 mM imidazole, 50 mM Arg, 50 mM Glu, 10% glycerol, Pierce universal protease, 1x protease inhibitor (Roche)), and lysed by sonication. After centrifugation, the supernatant was loaded onto a HisTrap HP column (5 mL, GE Healthcare) on an AKTA avant (GE Healthcare). The column was washed with 50 mL of buffer A (20 mM Tris, pH 8.0, 500 mM NaCl, 1 mM TCEP, 20 mM imidazole), and the protein was eluted with bufferA/buffer B (20 mM Tris, pH 8.0, 500 mM NaCl, 1 mM TCEP, 500 mM imidazole) gradient (100 mL). The protein was further purified by a HiTrap Heparin HP column (5 mL, GE Healthcare) and a HiLoad 16/600 Superdex 200 column (GE Healthcare). The protein was stored in buffer C (20 mM Tris, pH 8.0, 300 mM NaCl, 1 mM TCEP) at 19 mg/mL at −80 °C. The molecular weight of the protein was confirmed by LCMS (calc. 57455 Da, obs. 57457 Da). The typical yield of the protein is around 15 mg/L. For protein used in SPR, an Avi-tag (GLNDIFEAQKIEWHE) was inserted at the end of the gene resulting in a protein called PB2long-His-Avi. N510T mutation was introduced to the PB2long-His-Avi construct by Q5 SDM kit to generate PB2long-N510T-His-Avi. Both proteins were co-expressed with BirA and purified as described above.

### Crystallization, data collection, and structure determination

Crystals of PB2long were grown by hanging-drop vapor diffusion at 4 °C. The precipitant well solution consisted of 0.1 M MES pH 5.77, and 13.18% PEG 20,000. Crystals of the VX-787 complex were produced by soaking apo crystals in reservoir solution containing VX-787 at 1 mM final concentration. Crystals were cryo-protected by a harvesting solution containing reservoir solution supplemented by 20% glycerol and flash-cooled in liquid nitrogen for data collection. Datasets were collected under cryogenic conditions (100 K) at the Advanced Photo Source (APS) beamline 17-ID and Advanced Light Source (ALS) beamline 5.0.2. All data were processed using autoPROC, employing XDS for data integration and AIMLESS for scaling. Molecular replacement were carried out using PHASER with coordinates from PDB 2JDQ and 2VQZ representing the C-terminal domain and cap-binding domain, respectively. Iterative cycles of refinement and manual model building were carried out with PHENIX and COOT, respectively, until the models converged to acceptable levels of R factors and stereochemistry.

### PB2 fluorescence polarization assay

100 nM influenza A PB2cap or 1 nM influenza APB2long was added to a buffer containing 25 mM Tris, pH = 7.0, 150 mM KCl, 0.01% Tween-20, 1 mM EDTA and 1 mM TCEP. The inhibitor was pre-incubated with the protein for 30 minutes at room temperature. Binding equilibrium reactions were initiated by the addition of 100 nM probe for PB2cap or 5 nM probe for PB2long. Binding equilibrium was allowed to occur for 60 minutes at room temperature. The reactions were then read on a Perkin-Elmer EnVision 2101 reader (fluorescence polarization mode) using an excitation of 480 nm and emission of 535 nm.

### PB2 SPR binding assay

SPR analysis was performed on a Biacore T200 instrument at 15 °C at 60 µL/min in 50 mM Tris pH 7 with 150 mM KCl, 1 mM EDTA, 0.01% P20 and 1 mM TCEP. Protein stability was monitored with m^7^G, which has affinities of 94 and 86 µM for PB2long and PB2cap, respectively. Both PB2 proteins retained 100% activity over the course of 40 hours. Protein loading was 6000–7000 RU for both proteins. For control m^7^G, multi-cycle kinetics mode was used, which injects a concentration series and monitors association and dissociation for each injection. For the compound, the single-cycle kinetics mode was used, since dissociation times are long. This method injects a series of concentrations, and monitors association and dissociation for the series, but it does not require complete dissociation to baseline. All data analysis was performed in Biacore evaluation software.

### Cytopathic effect (CPE) inhibitory assay

MDCK cells in infection medium (DMEM with 0.1% BSA, 1% sodium pyruvate, 1% pen/strep) were seeded at 18,000 cells/well in 384-well plates and incubated for 24 hours at 37 °C and 5% CO_2_. Cells were infected using influenza virus A/H3N2/Udorn/72 at an MOI of 0.005 in the presence of TPCK trypsin (1 ug/mL) to promote viral spread. Compounds were added (16-point dose response, 0.5% final DMSO concentration) and the plates were incubated for 72 hours at 37 °C and 5% CO_2_. Cell viability was measured by adding Cell-Titer Glo (Promega) to the wells. Compound toxicity was evaluated in absence of virus infection using Cell-Titer Glo. The plates were read using the POLARstar Omega Microplate Reader.

### Influenza virus minigenome assays

293 T cells in Dulbecco’s modified Eagle’s medium (DMEM) minus phenol red, supplemented with 10% heat inactivated FBS, 1% sodium pyruvate and 1% L-glutamine (Cellgro, Manassas, VA) were transfected with pCAGGS expression vector encoding A/H3N2/Udorn/72 PB2, PB1, PA, NP and Influenza A Luciferase reporter plasmid with a 1:1:1:1:0.5 PB2:PB1:PA:NP with Fugene 6 transfection agent (Promega, Madison, WI) in OptiMEM (Gibco, Carlsbad, CA). Transfected cells were treated with compound 2 hours post-transfection, and plates were incubated for 48 hours at 37 C, 5%CO2. Compounds were prepared at 10 mM in 100% DMSO and added as a 1:3 8-point serial dilution with a 50 μM final concentration. Following incubation, cells were lysed and luciferase production quantified with the addition of Britelite Plus (Perkin-Elmer, Waltham, MA), with a 1:1 transfection mix:Britelite Plus ratio. CellTiter-Glo (Promega, Madison, WI) was added to cytotoxicity plates following manufacturer’s instructions to quantify cell survival.

## Electronic supplementary material

Supplementary material

PB2 primer sequences
