# Supplementary material for: Structural basis for therapeutic inhibition of influenza A polymerase PB2 subunit
Source: Sci Rep. 2017 Aug 24;7:9385. doi: 10.1038/s41598-017-09538-x (PMC5571044; doi:10.1038/s41598-017-09538-x)
Supplement: Supplementary file 1 — Supplementary material [file 41598_2017_9538_MOESM1_ESM.pdf]

Structural basis for therapeutic inhibition of influenza A polymerase PB2 subunit

Authors

Xiaolei Ma[1,4], Lili Xie[2], Charles Wartchow[1,4], Robert Warne[3], Yongjin Xu[4,#], Alexey Rivkin[4], David Tully[4], Steven Shia[1], Kyoko Uehara[2], Dianna M. Baldwin[5], Gladys Muiru[5], Weidong Zhong[5], Isabel Zaror[2], Dirksen E. Bussiere[1,4] and Vincent H. J. Leonard[5]

[1]Structural and Biophysical Chemistry, [2]Protein Sciences, [3]Virology Lead Discovery, [4]Global Discovery Chemistry and [5]Virology, Novartis Institutes for BioMedical Research, Emeryville, CA, USA. Correspondence should be addressed to xiaolei.ma@novartis.com or vincent.leonard@novartis.com

Supplementary materials:

Supplementary Figure 1. PB2 constructs and expression analysis.

Supplementary Figure 2. Close-up view of the complete PB2 active site in VX-787-bound form.

Supplementary Table 1. Solubility and purification analysis of selected PB2 constructs

Supplementary Table 2: PB2 primer sequences

Supplementary Figures

Supplementary Figure 1

a

Constructs in a 96-well plate:				the colored cells contain the starting site of each construct								
	1	2	3	4	5	6	7	8	9	10	11	12
A	3	12	21	34	40	44	54	60	67	70	73	76
B	85	88	101	108	116	130	138	143	152	158	170	176
C	196	198	209	211	215	217	219	222	225	230	232	234
D	236	241	247	249	251	257	262	266	268	275	277	288
E	290	292	301	305	309	318	241-537	241-759	268	290	268-759	234-759
F	3	12	21	34	40	44	54	60	67	70	73	76
G	85	88	101	108	116	130	138	143	152	158	170	176
H	196	198	236	241	215	217	219	222	225	230	232	234
	yellow cells: end at 741			red cells: negative clones			blue cells: end at 483					

b

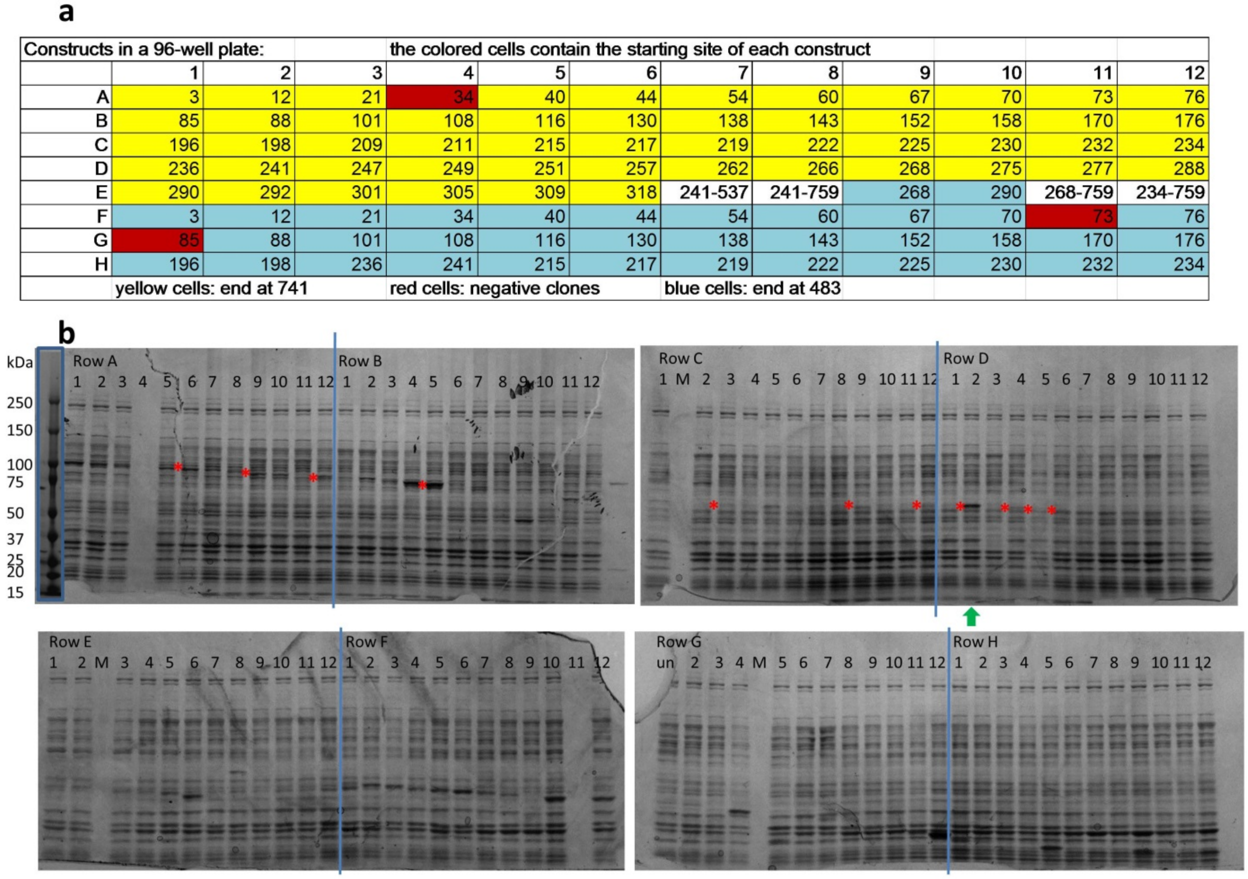

Supplementary Figure 1. PB2 constructs and expression analysis. (a) Schematic representation of the 96 PB2 truncations. The colored cells contain the starting site of each construct. Yellow cells have the residue ending at 741, whereas the blue cells have the residue ending at 483. The white cells contain both N- and C- termini. Total of 96 constructs were cloned and 93 of which were sequence confirmed. Three of the constructs PB2(34-741), PB2(73-483), PB2(85-483) did not result in positive clones, and are shown in red. (b) Coomassie blue-stained SDS-PAGE of the total lysate of each clone. The label corresponds to the well position in the above 96-well plate. A4 and F11 are negative clones therefore left blank. Label un means uninduced cells as negative control. M means protein marker which was transferred to membrane for western blot analysis (data not shown). The lanes with red asterisk were selected for further solubility and purification evaluation. The lane pointed by the green arrow is the final PB2long construct.

Supplementary Figure 2

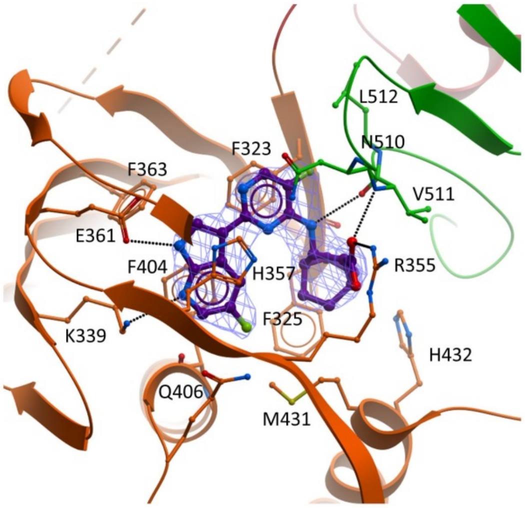

Supplementary Figure 2. Close-up view of the complete PB2 active site in VX-787-bound form. Active site residues and ligands are shown in ball-and stick mode; hydrogen bonds involving ligands are represented as dashed lines. The 2.5 Å 2|Fo| − |Fc| omit electron density is contoured at 3σ (blue mesh).

Supplementary Table 1. Solubility and purification analysis of selected PB2 constructs

Well position	Truncations	Expression	Solubility	Yield (mg/L)
A6	44-741	++	-	N/A
A9	67-741	++	-	N/A
A12	76-741	++	-	0
B5	116-741	+++	-	0
C3	209-741	+	-	0
C9	225-741	++	+	< 0.1
C12	234-741	++	++	3
D2	241-741	+++	+++	15
D4	249-741	++	+++	10
D5	251-741	+	++	6
D6	257-741	+	-	0

Supplementary Table 2: PB2 primer sequences

PB2 primers.xlsx
